# A New Approach to Modify Plant Microbiomes and Traits by Introducing Beneficial Bacteria at Flowering into Progeny Seeds

**DOI:** 10.3389/fmicb.2017.00011

**Published:** 2017-01-23

**Authors:** Birgit Mitter, Nikolaus Pfaffenbichler, Richard Flavell, Stéphane Compant, Livio Antonielli, Alexandra Petric, Teresa Berninger, Muhammad Naveed, Raheleh Sheibani-Tezerji, Geoffrey von Maltzahn, Angela Sessitsch

**Affiliations:** ^1^Bioresources, Center for Health & Bioresources, Austrian Institute of Technology GmbHTulln, Austria; ^2^Indigo Agriculture, CharlestownMA, USA

**Keywords:** seed, endophyte, microbiome, EndoSeed, flowers, strain delivery, application technology, *Paraburkholderia phytofirmans* PsJN

## Abstract

The microbial component of healthy seeds – the seed microbiome – appears to be inherited between plant generations and can dynamically influence germination, plant performance, and survival. As such, methods to optimize the seed microbiomes of major crops could have far-reaching implications for plant breeding and crop improvement to enhance agricultural food, feed, and fiber production. Here, we describe a new approach to modulate seed microbiomes of elite crop seed embryos and concomitantly design the traits to be mediated by seed microbiomes. Specifically, we discovered that by introducing the endophyte *Paraburkholderia phytofirmans* PsJN to the flowers of parent plants we could drive its inclusion in progeny seed microbiomes, thereby inducing vertical inheritance to the offspring generation. We demonstrated the introduction of PsJN to seeds of monocot and dicot plant species and the consequential modifications to seed microbiome composition and growth traits in wheat, illustrating the potential role of novel seed-based microbiomes in determining plant traits.

## Introduction

Plant internal microbiomes are complex communities of archaea, bacteria, and fungi, which live as endophytes in all plants (Turner et al., 2013; Hardoim et al., 2015). Their importance to plant growth and survival has recently been recognized much more extensively, following the series of revelations in humans about the far reaching importance of microbiomes for well-being and health. Those bacteria and fungi that live in the soil and rhizosphere of plants have received most attention (Philippot et al., 2013). They represent an important source of microorganisms, which are taken up by plant roots and further colonize the plant interior as endophytes (Hardoim et al., 2015). All plant organs have been found to host microbiomes. However, it is likely that it is the pre-existing microbiome of the planted seed that provides the foundation for successful plant growth, before being augmented by microbes from the soil. Seed microbiomes have not been studied extensively or defined until recently (Barret et al., 2015; Klaedtke et al., 2015). The microbiome typically found in seeds consists of a limited range of microbial species (Truyens et al., 2014). It appears to have evolved by co-selection with the plant species, providing important traits for plant survival (Puente et al., 2009; Johnston-Monje and Raizada, 2011; Hardoim et al., 2012). Its genes presumably complement those encoded in plant chromosomes and hence plant traits and evolution are determined by both plant and microbial genomes (Turner et al., 2013; Bouffaud et al., 2014; Delaux et al., 2014). The internal seed microbiome is inherited from the previous generation via the seed and so presumably consists of microbes that can survive desiccation and the conditions of seed storage (Truyens et al., 2014). Endophytic bacteria that colonize plants internally from the seed and promote growth and health are particularly useful in agricultural practice, because they escape competition with soil microorganisms and are in intimate contact with the plant tissues from an early stage on. The agriculturally relevant traits mediated by plant microbiomes imply that plant breeding needs to embrace creation of the best plant–microbiome associations for optimum plant performance. Here, we describe a new approach of introducing new microbes into seeds (EndoSeed^TM^) to modify the plant microbiome and plant traits in defined ways. It involves introducing a microbial strain into the parent plant before seed development is complete. The microbe subsequently becomes incorporated into seeds. After seed germination it multiplies and spreads through the new plant tissues of the next generation. As the external application of microbes, as it is practiced now, has many drawbacks, the approach presented here will have wide implications for plant breeding and the “design” of seeds, which have optimal plant–microbe associations.

Here, we demonstrated the feasibility of modifying seed microbiomes in a targeted, directed way by using the bacterium *Paraburkholderia phytofirmans* PsJN, which has been shown to be a very powerful plant growth promoter, with the ability to establish populations in a broad range of genetically unrelated plants. Colonization by strain PsJN results in increased plant growth and stress resistance in many plants including important crops and vegetables (summarized by Mitter et al., 2013). Recently, increasing efforts have been made to understand the molecular mechanisms of the interaction between strain PsJN and host plants. The observed positive effect of strain PsJN on plants is at least partly based on altered gene expression in the host plant (Lara-Chavez et al., 2015; Pinedo et al., 2015; Poupin et al., 2016) in the presence of PsJN. The bacterium itself is also active inside plant. More than 60% of all genes encoded on the genome were expressed in *P. phytofirmans* PsJN during endophytic colonization of potato plants and gene expression pattern changed in response to host plant drought stress (Sheibani-Tezerji et al., 2015). In shoots of *Arabidopsis thaliana* strain PsJN expresses genes related to iron storage and transport, which resulted in enhanced iron uptake and accumulation in the host plant (Zhao et al., 2016).

In this study, we showed that strain PsJN can be inserted into seeds of various crops including maize, wheat, soy, and pepper in the greenhouse as well as under field conditions. We determined by various microscopic tools that strain PsJN is localized in the seed embryo and strain specific TaqMan-qPCR was used to quantify the cells within seed. In field experiments, we tested PsJN-colonized seed and observed faster plant development as compared to control seed. For maize and wheat one should correctly use the term caryopsis instead of seeds. However, since the microorganisms are actually introduced into the seed, we use the concept seed in this paper for all plant species for the sake of simplicity.

## Materials and Methods

### Bacterial Strain and Inoculum Preparation

In this study, *Paraburkholderia* (formerly *Burkholderia*) *phytofirmans* PsJN (Sessitsch et al., 2005) and variants of PsJN chromosomally tagged with the beta-glucuronidase gene (Compant et al., 2005) were used. The bacterial strain was grown by loop-inoculating one single colony in LB broth (PsJN wild-type) and LB broth amended with spectinomycin (100 μg/mL) (PsJN::*gus*A110). Bacterial cultures were incubated at 28 ± 2°C for 2 days at 180 rpm in a shaking incubator. Cells in the stationary growth phase were harvested by centrifugation and the bacterial pellet resuspended by vortexing in 20 mL sterile PBS (0.2 g/L KCl, 1.44 g/L Na_2_HPO4, and 0.24 g/L KH_2_PO4, in dH_2_O, pH 7.4). The concentration of the suspensions was measured with a NanoDrop ND-1000 spectrophotometer (Wilmington, DE, USA) and adjusted to 3 × 10^8^ CFU/mL.

### Introducing *P. phytofirmans* PsJN into Seeds/Grains of Maize, Pepper, and Soy

Seeds of pepper (*Capsicum annuum* cultivar Feher) and soybean (*Glycine max* L. cultivars Merlin and Essor) were sown into potting soil in plastic trays and kept in a greenhouse chamber. Ten days after sowing seedlings were individually potted into 1L (soy), 3L (pepper) pots containing commercial potting soil. Soy plants were watered automatically twice a week by flooding for 10 min and fertilized once during the cultivation period with liquid fertilizer suspension Wuxal Super 3% (Aglukon) (NPK + trace elements). Pepper plants were watered daily and fertilized every 4 weeks with Wuxal Super 0.5%. The frequency of fertilizer application was increased to a weekly treatment upon fruit set of the pepper plants. Maize nursery was done as described elsewhere (Naveed et al., 2014). Specific inoculation of flowers was conducted when the plants reached growth stage 61–63 on the BBCH scale (Meier, 2001); for pepper and soy: first flower open – third flower open; for maize: flowering, anthesis. A suspension of *P. phytofirmans* PsJN and its variant *P. phytofirmans* PsJN::*gusA*, respectively and buffer only for the control were poured in pump spray bottles previously sterilized with 70% ethanol. Flowers were sprayed and a filter paper was used to shield the surrounding plant parts, such as leaves and stem from drift and to remove surplus inoculum to avoid dripping on the soil. The treated inflorescences/flowers were marked to allow for later identification. The inoculum was prepared as described above. Seeds or grains were sampled at maturity (maize and soy: 97 on the BBCH scale; pepper: 89 on the BBCH scale).

### Introducing *P. phytofirmans* PsJN into Spring Wheat Seeds under Field Conditions

The production of seeds internally colonized by *P. phytofirmans* PsJN under field conditions was tested with *Triticum aestivum* L (cultivar Trappe). Ten by 1.3 m plots were planted on March 13, 2014 with spring wheat at a density of 180 kg/ha in a field located in Tulln, Austria. Plants were sprayed with herbicide once (1.25 L/ha Andiamo Maxx) and fertilized twice. NPK-Fertilizer 16:6:18+5S was applied at a concentration of 300 kg/ha and N-Fertilizer 27% was applied at a concentration of 220 kg/ha. At flowering time, each plot was sprayed twice (June 4 and June 11, 2014) with a suspension of *P. phytofirmans* PsJN. The bacterial inoculant used for spraying was prepared as follows: endophytes were streaked on large (diameter: 14.5 cm) 20% tryptic soy agar plates, grown at 28°C for 2 days, scraped from the plates and suspended in 2L of 1x PBS supplemented with 20 g zeolite (used as a carrier) and 200 μL Silwet L-77 (final OD_600_ of about 0.1). Each plot was sprayed with 1 L of the corresponding treatment. Negative control plots were sprayed with 1x PBS containing zeolite and Silwet L-77. Seed was harvested at maturity (July 21, 2014, 97 on the BBCH scale) and used for further analysis.

### Detection and Quantification of *P. phytofirmans* PsJN in Plant Tissue by GUS-Staining and Cell Counting

Endophytic colonization of roots, stems, and leaves of maize plants by the *gus*A-labeled variant of *P. phytofirmans* PsJN was determined by plate counting and colonies were identified by comparison of the 16S-23S rRNA intergenic spacer region DNA fragment pattern to pure culture *P. phytofirmans* PsJN as described elsewhere (Naveed et al., 2014). Gus-staining of plant tissue was performed as following: The plant material was cut with a sterile scalpel and subsequently incubated in GUS-staining solution (1 mM EDTA, 5 mM potassium ferricyanide, 5 mM potassium ferrocyanide, 100 mM sodium phosphate, pH 7.0, 1% Triton-X-100, 0.1 mg/mL X-Gluc pre-dissolved in 5 μL/mg *N,N*-dimethylformamide, and 0.1% IPTG) directly after harvesting at 37°C for 20 h. Destaining was done by rinsing the samples with 70% ethanol. The ethanol was then discarded and the samples fixed in paraformaldehyde solution (4% paraformaldehyde dissolved in PBS at 60°C with constant stirring until clarifying of the solution) overnight at 4°C. Finally, the fixed samples were rinsed three times in PBS and stored in the last rinse at 4°C until further processing.

### Detection of PsJN in Seeds and Green Parts of Plants Using DOPE-FISH/CSLM Microscopy

For microscopy, plant samples were cut in 0.5 cm long sections. Samples were then fixed overnight at 4°C in a paraformaldehyde solution (4% in PBS, pH 7.2), and rinsed twice in PBS. Lysozyme solution (1 mg/mL in PBS) was then applied to the samples for 10 min at 37°C before being dehydrated in an ethanol series (25, 50, 75, and 99.9%; 15 min each step). Samples were included or not in LR white resin according to manufacturer’s instructions. Fluorescence *in situ* hybridization (FISH) using double labeling of oligonucleotide probes (DOPE-FISH) was carried out using probes from Eurofins (Germany) labeled at both the 5′ and 3′ positions. A probe mixture targeting eubacteria, composed of EUBmix (equivalent mixture of EUB338, EUB338II, and EUB338III) coupled with a ATTO488 fluorochrome (Amann, 1995; Daims et al., 2005) and a probe targeting the 23S rRNA gene of *P. phytofirmans* (5′-CTCTCCTACCATGCACATAAA3′) coupled with Cy5. The *P. phytofirmans*-specific probe was designed using Biosearch Technologies’ Stellaris FISH Probe Designer software^1^ and the sequence of chromosome 1 of strain PsJN. The probe was analyzed using NCBI, SILVA (Quast et al., 2012), probeCheck (Loy et al., 2007), mathFISH (Yilmaz et al., 2011), and Evaluation Tool^2^ and *in vitro-*validated using pure culture and suitable reaction settings (reaction temperature, formamide concentration, and probe concentration) for efficient hybridization. NONEUB probe (Wallner et al., 1993), coupled with Cy5 or ATTO488 was used independently as a negative control.

Fluorescence *in situ* hybridization was carried out at 46°C for 2 h with 10–20 μL solution (containing 20 mM Tris-HCl pH 8.0, 0.01% w/v SDS, 0.9 M NaCl, 10% formamide, and 10 ng/μL of each probe) applied to each plant sample placed on slides in a 50-mL moist chamber (also housing a piece of tissue imbibed with 5 mL hybridization buffer). Washing was conducted at 48°C for 30 min with a post-FISH pre-warmed solution containing 20 mM Tris-HCl pH 8.0, 0.01% (w/v) SDS, and NaCl at a concentration corresponding to the formamide concentration. Samples were then rinsed with distilled water before air drying for at least 1 day in the dark. The samples were then observed under a confocal microscope (Olympus Fluoview FV1000 with multiline laser FV5-LAMAR-2 and HeNe(G)laser FV10-LAHEG230-2). X, Y, Z pictures were taken at 405, 488, 633 nm and then merged (RGB) using ImageJ software. Z Project Stacks was then used to create pictures as described elsewhere (Campisano et al., 2014).

### DNA Isolation

Plant material was surface-sterilized as described earlier. Single surface-sterilized seeds were aseptically peeled using a scalpel, cut in pieces and crushed using a sterile mortar. Vegetative plant material was cut in pieces. All types of plant material were homogenized for 40 s in lysing matrix E (MPbio DNA isolation kit from soil) using a bead beater (FastPrep FP 120, Bio101, Savant Instruments, Inc., Holbrook, NY, USA). DNA was then extracted with the MPbio DNA isolation kit from soil (MP Biomedicals, Solon, OH, USA) according to protocol provided by the manufacturer. DNA (5 μl) was separated and visually tested for quality by electrophoresis (80 V) on 1% (w/v) agarose gels stained with ethidium bromide. DNA concentration was measured using a NanoDrop ND-1000 spectrophotometer.

### Quantification of *P. phytofirmans* PsJN in Plant Tissue Using qPCR

For quantification of *P. phytofirmans* PsJN in seeds and vegetative plant tissue we performed a qPCR using a Taqman probe and a Biorad CFX96 real-time detection system (Bio-Rad, Hercules, CA, USA). The probe and primers were designed in a previous study (Sheibani-Tezerji et al., 2015) to match the gene for transcription termination factor rho (Bphyt_1824) in the genome of strain PsJN. qPCR reactions contained (10 μl total volume): 1x SsoFast Probes, 0.5 μM of each primer, 0.35 μM probe, and 5–100 ng DNA. The qPCR was run at the following settings: hot start at 95°C for 2 min, 40 cycle denaturation at 95°C for 5 s and hybridization and elongation for 20 s at 59°C. For qPCR standard preparation, chromosomal DNA of strain PsJN was isolated as described above. DNA concentration was determined using a NanoDrop ND-1000 spectrophotometer and doing five replicate measurements. The mean value was used for further calculations. The number of DNA copies was calculated as follows:

$$\mathrm{number}\;\mathrm{of}\;copies=\frac{\mathrm{DNA}\;\mathrm{quantity}(g/\mu l)}{\mathrm{fragment}\;\mathrm{length}*660g/mol}*6,022*10^{\wedge }23.$$

Fragment length is 8,214,658 bp (size of PsJN genome). A dilution series was prepared to generate a standard curve. Unknown starting quantity of DNA copy numbers in the samples could be calculated based on the standard curve from the dilution series of known concentrations, which produced an *r*^2^ value of higher than 0.990. All data analysis was performed with Bio-Rad CFX Manager 3.0.

### Testing of the Effects of *P. phytofirmans* PsJN Incorporated in Seeds on the Development of Offspring Plants

(1) *Greenhouse experiments with spring wheat seeds:* plant nursery was done as described earlier. On day 17 after seed sowing, six plants per treatment were potted individually in pots with a diameter of 15 cm, containing commercial potting soil. Plant height was measured once a week and from day 48 onwards tillers were also counted. The appearance of the first spike on each plant was documented through day 73.

(2) *Field testing of spring wheat seeds:* the performance of seeds internally colonized by strain PsJN under field conditions was tested with *T. aestivum* L. (cultivar Trappe). Plots were planted on March 18, 2015 and field management was done as described above. Regular ratings of germination, plant height, tillering, and spike counting were performed and plant colonization by strain PsJN was tested by qPCR as described above.

### Analysis of Microbial Communities of Spring Wheat Seeds Colonized by Strain PsJN and Control Seeds Prepared in the Field

Genomic DNA was isolated using FastDNA SPIN Kit for soil as described above and concentration was adjusted to 5 ng/μl. A nested PCR approach was used to amplify bacterial 16S rDNA. The first amplification was performed with primers 799for (5′-AACMGGATTAGATACCCKG-3′) and 1392rev (5′-ACGGGCGGTGTGTRC-3′) (Chelius and Triplett, 2001) with the following reaction parameters: 25 μl reaction volume contained 200 nM of each primer, 300 μM dNTPs, 0.5 units KAPA HiFi DNA polymerase (Kapa Biosystems, Boston, MA, USA), 1x buffer, 5 ng template DNA. The amplification conditions were as follows: initial denaturation for 5 min at 95°C, 25x 30 s at 95°C, 30 s at 52°C and 30 s at 72°C, and a final elongation for 5 min at 72°C. PCR amplification was performed in a peqSTAR thermocycler (peQlab, Erlangen, Germany). Amplicons were subjected to electrophoresis (100 V for 1 h) in 2% (w/v) TBE agarose gels (Biozym Biotech Trading, Vienna, Austria). Amplification with the primer pair 799F and 1392R allows exclusion of the chloroplast 16S rDNA and results in co-amplification of bacterial and mitochondrial ribosomal genes, about 600 bp and 1,000 bp amplicon size, respectively. The band containing the PCR-product of bacterial 16S rDNA was excised. The gel pieces were put in a filter tip that was placed in a fresh tube and DNA was collected by centrifugation for 2 min at 1,000 rpm. The second amplification was performed with the primers 799for_illumina and 1175R1_illumina, using amplification reaction procedures as described above. PCR amplicons were subjected to electrophoresis and the 500 bp bands were excised and DNA collected as described above. Index PCR was performed with Nextera XT Index Kit (Illumina Inc., San Diego, CA, USA) according to the manufacturer’s protocol and resulting amplicons were purified using AMPure XP beads (New England Biolabs, Ipswich, MA, USA) following the manufacturer’s protocol. Amplicon concentration was measured using a Nanodrop and about 10 ng per sample were pooled. DNA quality and quantity of the pooled library was tested with an Agilent 2100 Bioanalyzer. The library denaturing, addition of internal control DNA (PhiX, Illumina) and sample loading were done according to the Illumina protocol. Sequencing was performed on a MiSeq desktop sequencer (Illumina Inc., San Diego, USA).

### 16S rRNA Gene Sequencing Processing

MiSeq raw data quality was checked in FASTQC (Andrews, 2010) and reads were screened for PhiX contamination using Bowtie 2.2.6 (Langmead and Salzberg, 2012). A Bayesian clustering for error correction (Nikolenko et al., 2013; Schirmer et al., 2015) was applied before merging the PE reads using PEAR 0.9.6 (Zhang et al., 2014) (*p* < 0.001). Forward and reverse primers were then stripped from merged reads employing Cutadapt 1.8.3 (Martin, 2011) and quality filtering performed in USEARCH v8.0.1517 (Edgar, 2013; Edgar and Flyvbjerg, 2015) (maximum expected error = 0.5). Filtered reads were labeled according to the sample name of origin and combined in QIIME (Caporaso et al., 2010). Sequences were dereplicated, sorted and clustered at 97% of similarity using VSEARCH 1.1.1 (Rognes, 2015). Chimeras were checked adopting both a *de novo* and a reference based approach, as routine of the above mentioned tool. The RDP classifier training set v15 (09/2015) was used as a reference database. METAXA2 (Bengtsson-Palme et al., 2015) was used to target the extraction and to verify the 16S V7-V9 region of the representative sequences. An optimal global alignment was applied afterwards in VSEARCH and a BIOM table generated. Taxonomy assignment was performed employing the naïve Bayesian RDP classifier (Wang et al., 2007) with a minimum confidence of 0.6 and a customized version of the Greengenes database (08/2013) (McDonald et al., 2012), including the PsJN strain sequence and taxonomy.

Community sequencing data are made available at NCBI SRA database under the accession SRP067570 and the BioProject number PRJNA305879.

### 16S rRNA Gene-Based Microbial Community Analysis and Statistics

An OTU-based analysis was performed in QIIME to calculate the richness and diversity after multiple rarefactions. The observed OTUs were counted and the diversity within each individual sample was estimated using the Simpson’s diversity index. Richness and diversity values were compared between the control and the treatment by means of permutational pairwise comparisons in the RVAideMemoire R package (Hervé, 2015). The resulting *P* values were adjusted by false discovery rate (FDR). Richness and diversity value boxplots were then plotted via ggplot2 (Wickham and Chang, 2015) package in R.

A data-driven adaptive method for selecting normalization scale quantile was conducted on the BIOM table and data normalized by scaling counts by the nth percentile of each sample’s nonzero count distribution in the metagenomeSeq Bioconductor package (Paulson et al., 2013; McMurdie and Holmes, 2014). The resulting normalized BIOM table was used for the beta-diversity analysis. Multivariate analysis of community structure and diversity was performed according to the recommendations by Anderson and Willis (2003): (1) unconstrained ordination offered by Principal Coordinate Analysis (PCoA), (2) constrained multidimensional scaling using Constrained Analysis of Principal Coordinates (CAP) as re-implemented in the vegan R package (Oksanen et al., 2015), (3) permutation test for assessing the significance of the constraints and permutational multivariate analysis of variance (PERMANOVA), and (4) individuation and correlation of OTUs responsible for shaping the diversity structure.

## Results

### Bacteria Colonize Seeds upon Flower Application

**FIGURE 1:**
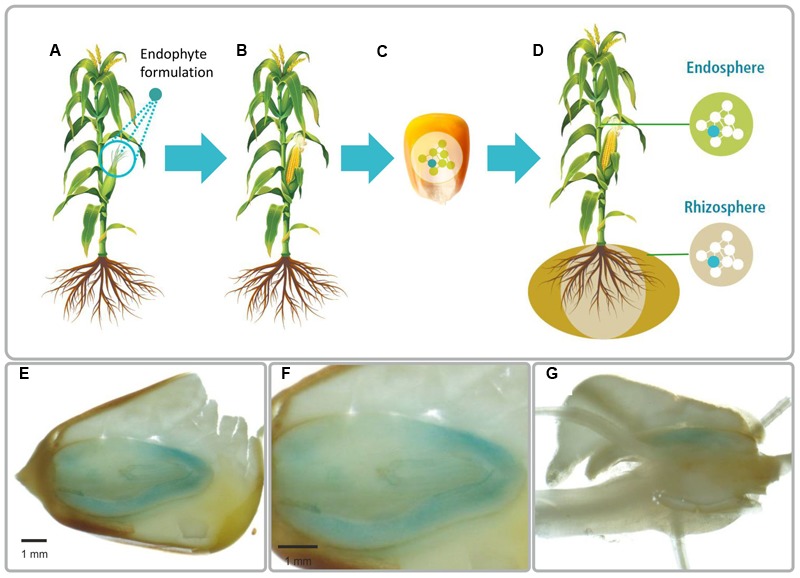
**Illustration of the method to introduce plant beneficial bacteria into plant seed (A–D). (A)** Plant flowers are sprayed with a bacterial suspension. **(B)** The bacteria colonize flowers and the developing seeds. **(C)** Mature seeds are collected and endophytes stay viable during seed storage. **(D)** Endophytes proliferate during germination and colonize the offspring plant generation. Light microscopy images of a mature maize colonized by *Paraburkholderia phytofirmans* strain PsJN::*gus*A **(E–G)**. The blue is due to GUS-stained bacterial cells. Strain PsJN is present inside the embryo **(E,F)** and in radicals **(G)**. PsJN starts moving from embryo to germinated parts **(G)**. In the picture **F**, we present a zoom in of the first photograph **(E)**.

The concept of introducing bacteria into plant seeds and consequently modifying the seed microbiome is illustrated in **Figure 1**. The bacteria are sprayed onto the parent flowers, enter the plant and colonize the emerging seeds. By planting the internally colonized seeds, the bacteria become activated and proliferate and colonize the offspring generation plant, thereby unfolding growth regulation effects from the first day of germination of the offspring crop generation. We demonstrated the feasibility of modifying seed microbiomes in a targeted, directed way by using the plant-growth promoting bacterium *P. phytofirmans* PsJN. Initially, a variant of strain PsJN chromosomally tagged with the beta-glucuronidase gene for detection and monitoring the strain by color formation (Compant et al., 2005) was either applied on seeds or sprayed on female flowers of maize (*Zea mays* L. cvs. Peso and Morignon) in the greenhouse. At maturity, we detected GUS-stained PsJN cells in maize seeds of plants that had been sprayed with the bacterium (**Figures 1E–G**) at viable population densities that ranged from 10^2^ to 10^5^ CFU/g fresh weight; no GUS-stained cells were recovered from control seeds from flowers sprayed with a solution lacking PsJN (not shown). Strain PsJN was not recovered from next generation seeds when the bacterium was applied on seed. After 12 months of storage of PsJN-colonized seeds, we still recovered about 100 viable cells per g maize seeds illustrating the stability of the bacterium in seeds.

### Bacterial Cells Are Located in the Seed Embryo

**FIGURE 2:**
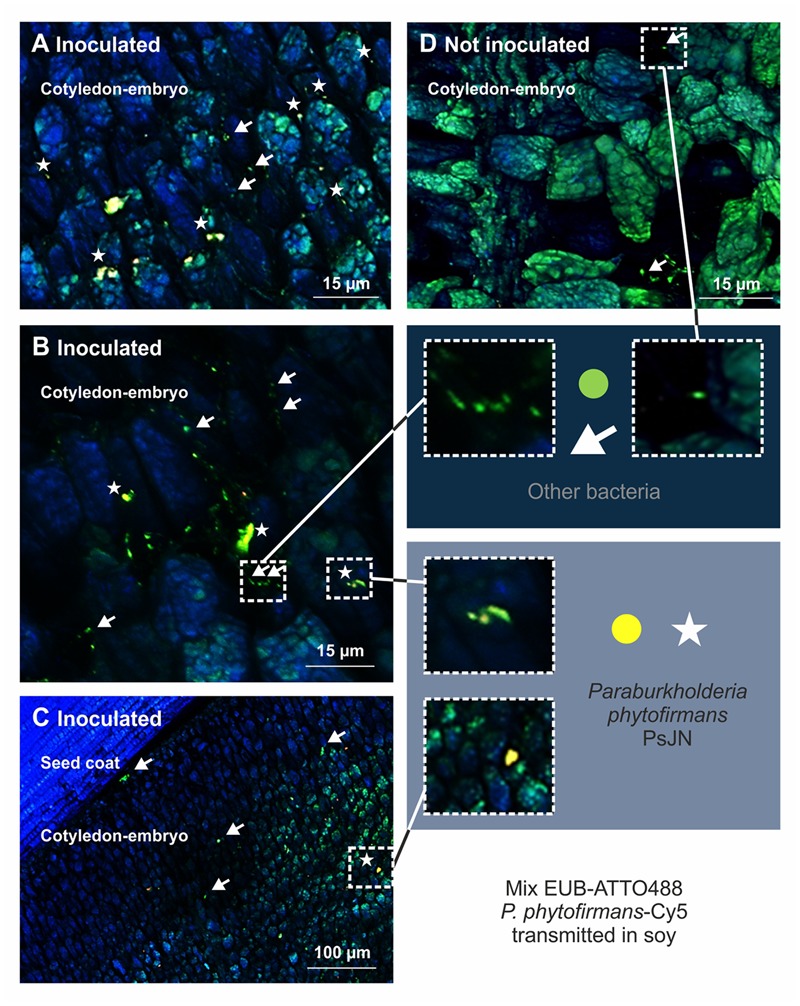
**Visualization of *P. phytofirmans* PsJN in seeds of *Glycine max* L. (soy) by DOPE-FISH/CSLM microscopy.** The mixEUB and *P. phytofirmans* probes were applied on mature seed from parent plants sprayed or not sprayed with strain PsJN. The presence of *P. phytofirmans* inside the embryo along with other microbes is shown **(A–C)**. *P. phytofirmans* cells were not detected in seeds of non-inoculated plants **(D)**. Seeds used for DOPE-FISH/CSLM microscopy were harvested 20 weeks after sawing (growth stage 97 on the BBCH scale).

We next evaluated whether the application of PsJN to flowers of dicotyledonous plants would result in colonization of their seed microbiomes and performed greenhouse experiments with soy (*G. max* L. cvs. Essor and Merlin) and pepper (*C. annuum* L. cv. Feher). We observed that PsJN was localized inside soy and pepper seeds by FISH using a specific probe targeting the 23S rRNA gene of *P. phytofirmans* and universal probes for bacteria. Yellow fluorescent PsJN cells were found inside the embryo of soy along with other bacteria. *P. phytofirmans* was detected in the cotyledon part of the embryo together with other bacteria (green fluorescent) (**Figures 2A–C**), which also colonized the seed coat (**Figure 2C**), while in control seeds only the native bacteria were present (**Figure 2D**). The NONEUB probe (negative probe not targeting bacterial sequences) was used to validate FISH on these samples and to exclude false positives (**Supplementary Figure S1**); only few natural green/blue-cyan-autofluorescent microbes could be seen inside the embryo of seeds colonized by PsJN and in control seeds (**Supplementary Figure S1**). The number of PsJN bacteria detected in soy seeds (tested by strain-specific qPCR) ranged from about 360 to about 4,500 genome equivalents per seed. Similar results were obtained with FISH microscopy of pepper seeds, and PsJN was detected within the embryo together with other bacteria that were also detected on the seed coat (**Supplementary Figure S2**).

### Field Application

The next step in our study was to test whether we can introduce bacteria and thereby modulate the seed microbiome during seed production in the field. We planted wheat (*T. aestivum* cv. Trappe) in an experimental field in Tulln (Austria). At flowering we applied *P. phytofirmans* PsJN. At seed maturity, we found strain PsJN to be effectively introduced into the seeds – 21 out of 24 seeds tested positive in PsJN-specific qPCR assays. This means that up to 92% of wheat seeds became colonized by strain PsJN after spraying of young parent flowers. The number of genome equivalents per seed varied strongly (347.5 ± 182.5).

### Changes in Plant Traits

**FIGURE 3:**
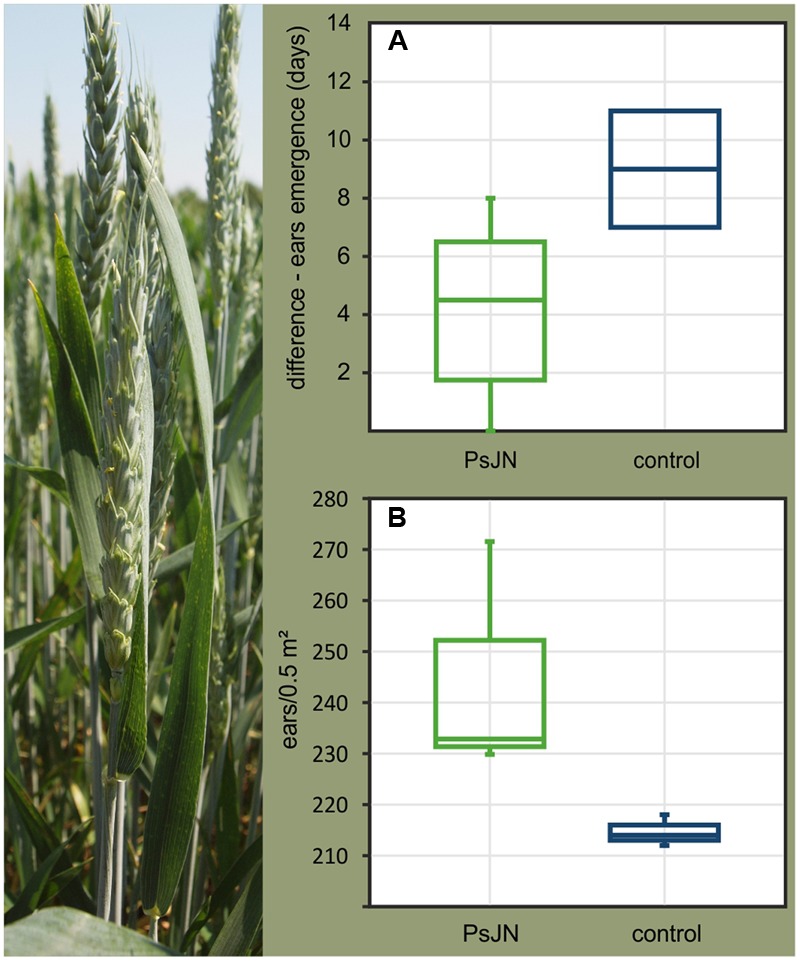
**Differences in ear emergence times between wheat plants (*Triticum aestivum* cv.** Trappe) growing from seeds colonized by *P. phytofirmans* PsJN and control seeds **(A)** observed in greenhouse pot experiments and in the field **(B)**. **(A)** The different time points of flowering in control and PsJN-colonized plants is shown. **(B)** A significantly higher number of ears per square meter in the plots was observed for PsJN-plants (*n* = 722) as compared to control plants (*n* = 644) (*F*-test; *p* = 0.018). All wheat plants tested belonged to the F1 generation, derived from parent plants which were sprayed with a suspension of *P. phytofirmans* PsJN or sterile buffer (control).

One of the main purposes of modulating seed microbiomes is to achieve improvement of a desired agricultural trait such as growth enhancement of the offspring plant. Therefore, we compared the growth and development of wheat plants growing from seeds internally colonized by *P. phytofirmans* PsJN in pot experiments in the greenhouse as well as in the field with plants growing from non-colonized control seeds. Seeds were stored for 2 and 7 months at room temperature before being planted in the greenhouse and field, respectively. In our greenhouse experiments plants emerging from PsJN-colonized seeds showed significant alterations in spike onset, which started an average of 5 days earlier in PsJN-plants than in plants emerging from control seeds (**Figure 3A**). Similar effects were observed in the field. Ninety-four days after sowing (plants reached growth stage 69 on the BBCH scale; end of flowering), the number of ears per square meter in the field was significantly higher in plants emerging from PsJN-seeds as compared to the control plants owing to the earlier flowering (**Figure 3B**). Further, whatever the variation in numbers of PsJN in the planted seeds, the effects on the crop were relatively uniform and essentially non-overlapping with the controls.

Colonization of offspring plants by seed-borne PsJN was tested by strain-specific qPCR. In field grown wheat plants we detected PsJN in root and shoot tissue with an average of 981 ± 738 and 500 ± 357 genome equivalents per gram plant tissue. Passage of strain PsJN from colonized seeds to the next generation of seeds was tested for pepper, soy, and wheat but PsJN was not found in any of the seeds.

### Changes in the Seed Microbiome

**FIGURE 4:**
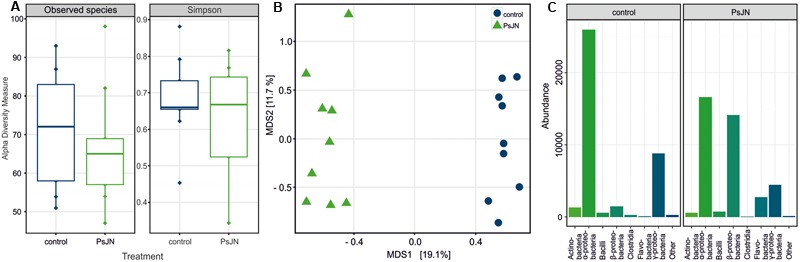
**Seed endophyte community profiling based on 16S rRNA gene V5–V7 sequences.** Bacterial communities in wheat (*T. aestivum* cv. Trappe) seeds colonized by *P. phytofirmans* PsJN and control seeds were compared. **(A)** Alpha diversity within subject by treatment (seeds colonized by PsJN and control seeds), as measured counting the observed OTU richness (Observed) and calculating the Simpson’s diversity index (Simpson). A permutation pairwise comparison (RVAideMemoire R package) showed (*p* > 0.05, perm = 9999) that neither the richness nor the diversity values were significantly different when grouped by treatment. **(B)** Bray–Curtis beta diversity among subjects as depicted by a Principal Coordinates Analysis (PCoA). Control samples are shown in blue, PsJN-colonized seed samples in green **(A,B)**. A permutation test assessed the significance of the treatment on a Constrained Analysis of Principal Coordinates (CAP) (*p* < 0.001, perm = 9999) (vegan R package). **(C)** Proportional abundance barplot of the most variant OTUs by treatment summarized at class level. The OTUs were determined after univariate comparison of OTU abundance between control and treatment (Mann–Whitney–Wilcoxon, *p* < 0.05 FDR corrected) (RVAideMemoire R package) and plotted using the phyloseq R package.

For a comprehensive assessment of the effects of incorporating selected bacteria into wheat seed on the bacterial seed microbiome we performed a culture-independent community analysis of single seeds by Illumina 16S rRNA gene-amplicon sequencing. Nine replicates of control seeds (plants were sprayed with sterile buffer) and PsJN-seeds (plants were sprayed with PsJN) were used for sequencing (sequencing statistics are given in Supplementary Table S1). The seed bacterial communities were dominated by Proteobacteria, which made up 92 and 90% of the OTUs in the control seeds and PsJN-seeds, respectively. While the species richness and diversity (α-diversity, **Figure 4A**) were not affected by the introduction of strain PsJN into spring wheat seeds, the sequencing results showed a clear effect on the community structure (β-diversity, **Figure 4B**). The treatments produced differences mainly in the abundance of certain groups (**Figure 4C**). Besides the expected increase in β-Proteobacteria by the introduction of strain PsJN (4% in control seeds and 39% in PsJN-seeds), Flavobacteria were enriched in PsJN-colonized seeds, whereas OTUs belonging to α-Proteobacteria decreased upon introduction of PsJN (**Figure 4C**). Flavobacteria constituted less than 0.4% in control seeds but were enriched to 6% in PsJN-seeds and the α-Proteobacteria share was reduced from 67% of the bacteria in control seeds to 39% in PsJN-colonized seeds (**Figure 4C**).

## Discussion

Our data show that plant beneficial bacteria can be selectively introduced into plant seeds and replicated to a relatively high cell number and so provided to the next generation of plants. Along with introducing a selected bacterium, changes in the composition of the seed microbiome were induced. These modifications to the seed microbiome composition were likely determined in the parent plant in and around the tissues that give rise to gametes and seeds during grain filling.

Changes in the composition of endophytic microbiomes as a result of infection with selected bacteria have been shown for vegetative plant organs (Reiter et al., 2002; Andreote et al., 2010; Ardanov et al., 2012) and correlated with disease resistance in potato (Ardanov et al., 2012). The microbiome of plants has been recognized as an essential component of the holobiont plant and the sum of all genomes of the microbiome together with the host genome make up for the hologenome of plants, which determines plant fitness (Jefferson, 1994). Consequently, changes in the plant microbiome would create new holobionts and modulate the behavior of the host (Turner et al., 2013). The introduction of strain PsJN into spring wheat seed caused changes in the microbiome composition, thus the observed effects on plant growth could be at least partly mediated through the activity of other members of the microbiome.

Our approach opens up new ways to explore links between inherited microbiome constituents and plant traits. Such effects can be via the added microbe alone or via the observed community effects. In our study, introduction of *P. phytofirmans* PsJN induced shifts in the endophytic bacterial community in wheat seeds and offspring plants showed earlier spike onset compared with non-treated plants. Such effects on flowering were not unexpected as it is known that *P. phytofirmans* PsJN speeds up maturity in many of its host plants and induces an earlier start in flower formation (Poupin et al., 2013; Wanga et al., 2015). In *A. thaliana* alterations in anthesis correlated with an earlier induction of flowering control genes in PsJN-inoculated plants as compared to control plants (Poupin et al., 2013). This observation points to a direct effect of the inoculant strain on host plant behavior. An indirect effect due to shifts in the community, however, cannot be fully excluded as the number of PsJN cells in plant tissue was low (few hundred genome equivalents per gram plant).

Following application on flowers *P. phytofirmans* PsJN colonized the embryo of seeds of both principle routes of seed colonization via flowers appear possible: (1) penetration of the nectarthodes (Pusey, 2000; Pusey and Curry, 2004); or alternatively, (2) settling on the stigma, which then enables further colonization along the style, finally leading to introduction of the bacteria into the ovary (Pusey and Curry, 2004). The stigma/style-pathway for seed colonization by strain PsJN is supported by our experiments with soy. The efficiency of introducing PsJN differed strongly in the two soy varieties tested – 50% of seeds of cultivar Essor contained PsJN but only 17% of cultivar Merlin. The two soy cultivars tested differ in the maturity, with Essor being early (00) and Merlin very early to mature (000). The flowers of both cultivars were sprayed on the same day. Differences in the flower age and condition of the stigma as well as the advance of the fertilization process could thus have influenced the susceptibility of soy flowers to invading PsJN cells.

The relative ease of introducing bacteria into plant seed by applying them on flowers of parent plants indicates that at least a part of the seed microbiome may derive from flower or pollen colonizing microorganisms (Aleklett et al., 2014; Ushio et al., 2015) and the air or insects visiting the plant during flowering might be other important source for seed endophytes. This aspect has not yet been studied in detail.

The use of microbial inoculants in crop production and protection is a rapidly growing area in agricultural technology. Growing environmental and human health concerns over the use of agrochemicals in plant protection and nutrition stress the urgent need for biological alternatives that can increase and sustain production while maintaining ecosystem functioning and securing vital resources. However, to realize large-scale implementation of microbial strains in agricultural practice we need to develop strategies for successful delivery of beneficial microbes into the plant under field conditions. Such strategies are largely missing (especially for gram-negative bacteria) and this represents a bottleneck in practical application. Our study is breaking new ground in this regard by targeting plant reproductive organs as entry ports and using the plant seed as a protective carrier for microbial inoculants.

By the approach presented here bacteria can be stably integrated into seeds and upon germination the integrated endophytes may proliferate and colonize the offspring generation plants. The introduction of plant beneficial bacteria into seed has many advantages over an external application by, e.g., seed coating. We assume, that inside the seed, the inoculant strain is more protected from competition with other microbes than in the rhizosphere and soil. Moreover, it might be less susceptible to environmental and mechanical perturbation and it is better compatible to chemical seed coatings and thus can be applied in combination with conventional seed treatments. In this study, we recovered viable cells of *P. phytofirmans* PsJN from maize seeds after 1 year of storage, demonstrating a shelf-life in line with the requirements on storage stability in agricultural practice.

## Conclusion

The presented approach for the delivery of plant beneficial microbes opens up new avenues in crop breeding by providing a means to introduce new traits into plants within one generation without the need of plant genetic manipulation and to design novel strategies to deliver viable bacteria into plants to overcome current limitations in crop production.

## Author Contributions

BM and AS conceived and designed the research. NP, LA, and BM analyzed data. NP performed DNA isolation, PCR and qPCR, plant experiments and designed figures. MN performed plant experiments and microscopy. SC and TB designed FISH probe and performed FISH experiments. AP performed illumina amplicon sequencing. LA analyzed sequencing data. RS-T developed the qPCR system. BM, RF, and GvM wrote the paper.

## Conflict of Interest Statement

The authors declare that the research was conducted in the absence of any commercial or financial relationships that could be construed as a potential conflict of interest.

## Footnotes

1 www.biosearchtech.com/stellarisdesigner/

2 www.arb-silva.de/search/testprobe/

## Supplementary Material

FIGURE S1 **Negative control of DOPE-FISH/CSLM microscopy on seeds of *Glycine max* L. (soy) containing *P. phytofirmans* PsJN.** The use of the NONEUB probe does not show bacteria except for a few autofluorescent microbes **(A–D)**. Seeds used were collected from fully ripe fruits (growth stage 89 on the BBCH scale).

FIGURE S2 **Visualization of *P. phytofirmans* PsJN in seeds of *Capsicum annuum* L. (pepper) by DOPE-FISH/CSLM microscopy showing the presence of *P. phytofirmans* (yellow) inside the embryo together with other bacteria (green) (A,B)**.
