# Supplementary figures and images for: A New Approach to Modify Plant Microbiomes and Traits by Introducing Beneficial Bacteria at Flowering into Progeny Seeds
Source: Front Microbiol. 2017 Jan 23;8:11. doi: 10.3389/fmicb.2017.00011 (PMC5253360; doi:10.3389/fmicb.2017.00011)

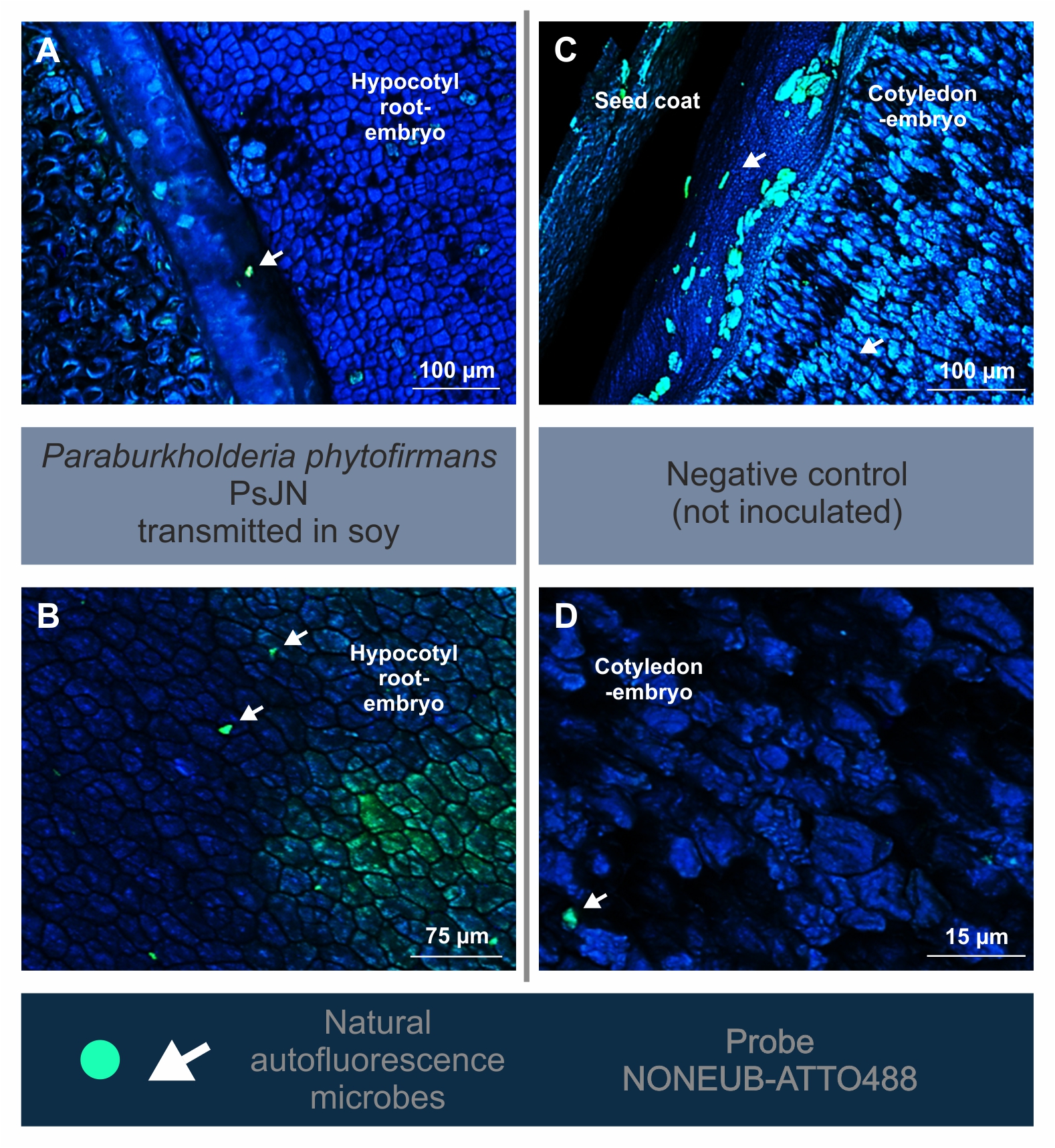

Supplement: FIGURE S1 — Negative control of DOPE-FISH/CSLM microscopy on seeds of Glycine max L. (soy) containing P. phytofirmans PsJN. The use of the NONEUB probe does not show bacteria except for a few autofluorescent microbes (A–D). Seeds used were collected from fully ripe fruits (growth stage 89 on the BBCH scale). [file Image_1.JPEG]

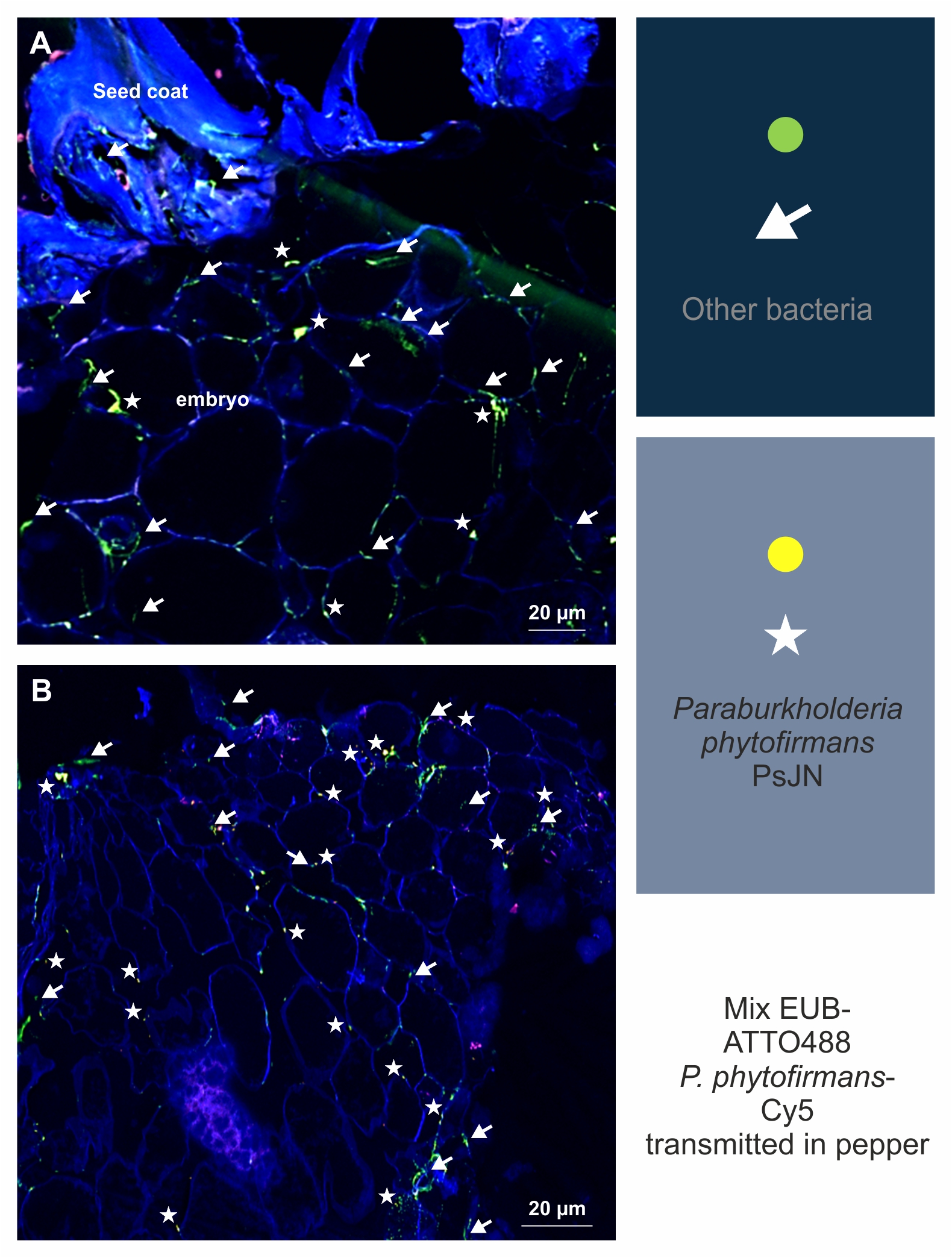

Supplement: FIGURE S2 — Visualization of P. phytofirmans PsJN in seeds of Capsicum annuum L. (pepper) by DOPE-FISH/CSLM microscopy showing the presence of P. phytofirmans (yellow) inside the embryo together with other bacteria (green) (A,B). [file Image_2.JPEG]
