# Supplementary material for: A New Approach to Modify Plant Microbiomes and Traits by Introducing Beneficial Bacteria at Flowering into Progeny Seeds
Source: Front Microbiol. 2017 Jan 23;8:11. doi: 10.3389/fmicb.2017.00011 (PMC5253360; doi:10.3389/fmicb.2017.00011)
Supplement: Supplementary file 3 [file Table_1.docx]

| **SI Table 1 16S rRNA gene amplicon sequencing statistics** | | |
| --- | --- | --- |
|  | control seeds | PsJN-seeds |
| total read count | 1,089,120.00 |  |
| average read counts | 11,203.33 ± 7,752.89 | 32,191 ± 20,791.57 |
| average read length | 367.18 ± 31.55 bp |  |
| average OTUs | 89.44 ± 12.53 | 127.22 ± 37.18 |
| **Alpha-diversity** | | |
| observed OTUs | 74.2 ± 12.30 | 65.58 ± 15.70 |
| Simpson's diversity index | 0.69 ± 0.12 | 0.64 ± 0.15 |
| The average read counts, average read length, average number of OTUs and alpha-diversity characteristics are shown as mean ± s.d. of nine replicates per treatment (control, seeds emerging from plants sprayed with PsJN). | | |
